# Hypnotism and Its Application to Practical Medicine

**Published:** 1897-12

**Authors:** 


Hypnotism and its Application to Practical Medicine. By Otto Georg Wetterstrand, M. D., Member of the Society of Swedish Physicians at Stockholm, etc., etc. Authorised translation, by Henrik G. Petersen, M. D. Together with medical letters on hypno-suggestion, etc. Octavo, pp. 183. New York: G. P. Putnam's Sons, 27 West Twenty-third street. 1897.

This well-published book is a fair presentation of the subject as it is taught and practised at Nancy, France. The author is a disciple of Bernheim and has followed his lead. He gives us his results in cases of insomnia, habitual headache, neuralgia, paralyses of organic origin, locomotor ataxia, epilepsy, chorea, stuttering, neurasthenia, hysteria, drug habits, rheumatism, hemorrhages, consumption, asthma, heart diseases, diseases of the stomach, diarrhea, Bright's disease, incontinence of urine, children's diseases, menstrual disturbances, external diseases, like sprains and synovitis, and in obstetrics.

He concludes, after citing a number of illustrative cases, that the hypnotic treatment is of great and lasting benefit and often the only means that can secure the desired result—recovery of health.

This work would have attracted much more attention if it contained material that was new to the profession. As it is, there is not much in this book that is not found in Herter's translation of Bernheim's work, which appeared eight years ago.

In this country hypnotic phenomena have been carefully studied and observed and no such results are obtainable in our clinics as are seen in Europe. The psychic life is different here and we must not expect results of much therapeutic value. J. W. P.

